# Longitudinal Macro/Microstructural Alterations of Different Callosal Subsections in Parkinson’s Disease Using Connectivity-Based Parcellation

**DOI:** 10.3389/fnagi.2020.572086

**Published:** 2020-11-04

**Authors:** Jingjing Wu, Tao Guo, Cheng Zhou, Xiaojun Guan, Ting Gao, Min Xuan, Quanquan Gu, Peiyu Huang, Zhe Song, Jiali Pu, Yaping Yan, Jun Tian, Baorong Zhang, Xiaojun Xu, Minming Zhang

**Affiliations:** ^1^Department of Radiology, The Second Affiliated Hospital, School of Medicine, Zhejiang University, Hangzhou, China; ^2^Department of Neurology, The Second Affiliated Hospital, School of Medicine, Zhejiang University, Hangzhou, China

**Keywords:** Parkinson’s disease, corpus callosum, subsection, connectivity-based parcellation, diffusion tensor imaging

## Abstract

**Background:**

The corpus callosum (CC) is an important feature of Parkinson’s disease (PD) not only in motor but also in non-motor functions. However, CC is not a homogeneous component, and the damage of specific subsection may contribute to corresponding clinical deficit.

**Objective:**

The objective of the study is to investigate the structural alterations of different callosal subsections cross-sectionally and longitudinally in PD and evaluate their relationships to clinical performance.

**Methods:**

Thirty-nine PD patients who had been longitudinally reexamined and 82 normal controls (NC) were employed. According to their specific callosal–cortical connectivity, 3D CC was divided into five subsections (including prefrontal, premotor, motor, somatosensory, and temporal–parietal–occipital subsection). The fractional anisotropy (FA), mean diffusivity (MD), and volume of whole CC and its subsections were computed and compared between groups. Regression model was constructed to explore the relationships between callosal structure and clinical performance.

**Results:**

At baseline, PD did not show any significant macro/microstructural difference compared with NC. During disease course, there was a decreased FA and increased MD of whole CC as well as its subsections (except temporal–parietal–occipital subsection), and the volume of motor subsection was decreased. Moreover, the FA of temporal–parietal–occipital subsection and volume of motor subsection were correlated with the mood domain at baseline, and the MD of somatosensory subsection was associated with the motor domain at follow-up.

**Conclusion:**

The structure of CC and its connectivity-specific subsections remain preserved at a relatively early stage in PD and are progressively disrupted during disease course. Besides, different callosal subsections possess specific associations with clinical performance in PD.

## Introduction

Parkinson’s disease (PD) is widely known as a slowly progressive movement disorder, which is demonstrated with complex clinical symptoms including classic motor dysfunctions (Lees et al., 2009) and various non-motor disturbances (Jankovic, 2008). Previous neuroimaging studies have identified the involvement of the corpus callosum (CC) to be an important feature of PD not only in motor but also in non-motor functions (Chan et al., 2014; Goldman et al., 2017). CC is the largest bundle between hemispheres (Hofer and Frahm, 2006) and participates in the mediation of multifunctions (Fabri et al., 2011, 2014) through structural connectivity or functional activation. Specifically, CC is not a homogeneous component, the fibers of which maturate at different ages and have the distinct vulnerability to pathological changes (Aboitiz et al., 1992; Aboitiz and Montiel, 2003), indicating the existence of different fiber compositions (Fabri et al., 2014). Indeed, the alterations of callosal subsections differed among PD patients with different cognitive levels (Goldman et al., 2017; Bledsoe et al., 2018) and patients with different subtypes (Chan et al., 2014). Given that the different activation loci were consistently detected in discrete CC regions according to peripheral stimuli (Fabri et al., 2011) and the connections of distinct callosal subsections to cortices were differential (Caille et al., 2005), the damage in a specific callosal subsection may contribute to the corresponding clinical deficit in PD.

On structural magnetic resonance imaging (MRI), PD shows callosal atrophy (Goldman et al., 2017; Lenka et al., 2017). The volume includes macrostructural information, which could elucidate the morphological change in PD. Besides, diffusion tensor imaging (DTI), which is sensitive to the microstructural alterations in white matter tracts and allows *in vivo* reconstruction of white matter tracts based on the directional diffusion properties of water, has also revealed differential callosal abnormalities across PD (Goldman et al., 2017; Bledsoe et al., 2018). Though a growing number of structural MRI and DTI studies explored PD, little is known about the co-appearance of macrostructure and microstructure in topographically defined callosal subsections and their relationships with clinical performance in PD. Moreover, to date, longitudinal studies in PD on structural alterations of callosal subsections over time have not been reported. Monitoring callosal fiber with imaging structural metrics could non-invasively explore the callosal alterations in PD. Connectivity-based parcellation is a method that separates the whole CC into functionally relevant subsections for precise mapping of its boundaries within subjects, and it better reflects the functional meaning of callosal subsections (Huang et al., 2005; Hofer and Frahm, 2006). Thus, it would precisely reflect the alterations of topographically defined callosal subsections in abnormal conditions and provide a novel insight into investigating their relationships with clinical performance in PD.

This study aimed to clarify the structural alterations in topographically defined callosal subsections cross-sectionally and longitudinally in PD and evaluate their relationships to clinical performance.

## Materials and Methods

### Subjects and Clinical Domain Calculation

The subjects were recruited according to the following criteria in this study: (a) had no history of other psychiatric or neurologic disorders or brain trauma, (b) had no general exclusion criteria for MR scanning, (c) were right handed, (d) signed the informed consent forms in accordance with the approval of the Medical Ethic Committee of The Second Affiliated Hospital of Zhejiang University School of Medicine, and specifically for PD patients, (e) diagnosed as PD by an experienced neurologist (BRZ) according to UK Parkinson’s Disease Society Brain Bank criteria (Hughes et al., 1992), and (f) had a follow-up 3-T MRI scanning and clinical evaluation. As a result, 39 PD patients with a mean follow-up time interval of 21 months and 82 age-, gender-, and education-matched normal controls (NC) were recruited. All procedures performed in this study involving human subjects were in accordance with the ethical standards of the institutional and national research committee and with the 1964 Helsinki Declaration and its later amendments or comparable ethical.

Magnetic resonance imaging scanning and clinical evaluations including age, gender, education, disease duration (from the day that parkinsonian symptoms occur to image scanning), Unified Parkinson’s Disease Rating Scale (UPDRS), Hoehn–Yahr Scale (H-Y), Mini-Mental State Examination (MMSE), Hamilton Depression Scale (HAMD), Hamilton Anxiety Scale (HAMA), Epworth Sleepiness Scale (ESS), and Parkinson’s disease sleep scale (PDSS) were acquired. For PD patients taking anti-parkinsonian drugs, all examinations were carried out after withdrawing all anti-parkinsonian medicine overnight (at least 12 h) to make sure they were in “OFF” status, and the total daily levodopa equivalent dose (LED) was recorded.

Given the discrepancy of scoring rules and ranges among different clinical scales, we transformed each scale to its *z*-score. The formula of *z*-score was as follows:

$$z_{\text{k}}=\left(\mathrm{crude}\mathrm{score}-\text{mean}\right)/\mathrm{standard}\mathrm{deviation}\left(\text{SD}\right)$$

Of note, because the more severe the disease was, the lower the PDSS and MMSE score were, we transformed its *z*-score to its negative number first. Then, the motor domain was assigned as the *z*-score of UPDRS III, the mood domain was the mean of the *z*-score of HAMD and HAMA, sleep domain was the mean of *z*-score of ESS and the negative *z*-score of PDSS, and cognition domain was the mean of the negative *z*-score of MMSE, and then, for measuring clinical progression, the annualized clinical change of each domain was calculated as {[(follow-up - baseline)/time interval]}.

### MRI Data Acquisition

Subjects were scanned on a 3.0 T MRI machine (GE Discovery 750) equipped with an eight-channel head coil. During MRI scanning, their heads were fixed with foam pads, and earplugs were provided to reduce the noise.

Structural T1-weighted images were acquired using a fast spoiled gradient recalled sequence: repetition time = 7.336 ms, echo time = 3.036 ms, inversion time = 450 ms, flip angle = 11°, field of view = 260 × 260 mm^2^, matrix = 256 × 256, slice thickness = 1.2 mm, voxel size = 1.02 × 1.02 × 1.2, and 196 continuous sagittal slices.

Diffusion tensor imaging images were scanned using a spin-echo echo-planar imaging sequence with 30 gradient directions (*b* value = 1,000 s/mm^2^): TR = 8,000 ms, TE = 80 ms, flip angle = 90°, field of view = 256 × 256 mm^2^, matrix = 128 × 128, slice thickness = 2 mm, voxel size = 2 × 2 × 2, slice gap = 0 mm, and 67 interleaved axial slices.

T2-weighted images and spin echo-inversion recovery sequence (FLAIR) images were additionally acquired and then visually examined by an experienced radiologist for excluding patients with vascular disease, traumatic disease, or tumor. The parameters were as follows: T2: fast spin echo sequence, repetition time = 3,000 ms, echo time = 106.7 ms, flip angle = 90°, field of view = 240 × 240 mm^2^, matrix = 512 × 512, slice thickness = 4.0 mm, voxel size = 0.47 × 0.47 × 4, and 38 continuous sagittal slices; FLAIR: repetition time = 11,000 ms, echo time = 157.528 ms, inversion time = 2,250 ms, flip angle = 90°, field of view = 220 × 220 mm^2^, matrix = 256 × 256, slice thickness = 4.0 mm, voxel size = 0.86 × 0.86 × 4, and 42 continuous sagittal slices.

### DTI Preprocessing and Seed Definition

Diffusion tensor imaging images were preprocessed by the Pipeline for Analyzing braiN Diffusion imAges toolbox (PANDA_1.3.1_64^1^) (Cui et al., 2013), which incorporates FMRIB Software Library (FSL^2^) and Diffusion Toolkit software^3^. The preprocessing procedures were as follows: (1) brain extraction using FSL’s “BET” tool with factional intensity threshold = 0.25; (2) eddy current-induced distortion and head motion artifacts correction using FSL’s “eddy_correct” tool; after which, the original b-vectors were rotated according to the affine transformation; and (3) DTI metrics [fractional anisotropy (FA); mean diffusivity (MD)] fitting using FSL’s “DTIFIT” tool (with standard linear regression). To ensure the accuracy of brain extraction, each extracted brain was scrutinized by an experienced radiologist.

**FIGURE 1:**
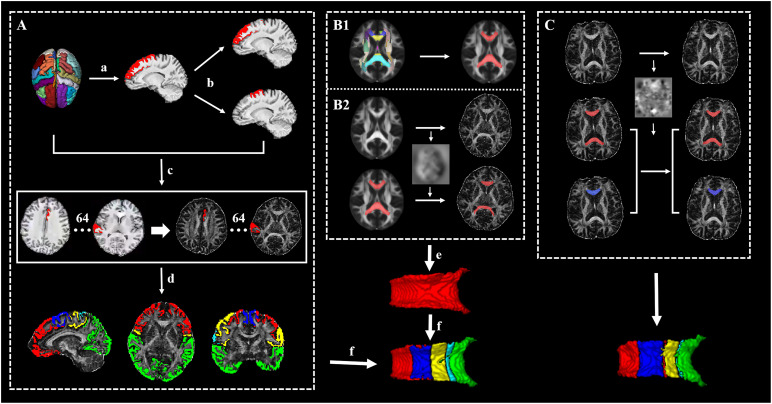
The framework of the whole processing steps. **(A)** The generation of targets: **(a)** The cortex was segmented to 62 segmentations by using the Desikan–Killiany–Tourville (DKT) cortical labeling protocol, then thresholded with 0.5, and binarized. **(b)** The premotor gyrus and prefrontal gyrus were separated from the superior frontal gyrus. **(c)** A total of 64 cortical segmentations were acquired and transformed to the diffusion space. **(d)** A total 64 cortical segmentations were merged to five distinct segments. **(B1)** The acquisition of standard callosal mask. **(B2)** The semi-automatic procedure of seed delineation. **(e)** Manual adjustment of seed. **(f)** Connectivity-based parcellation. **(C)** The segmentation scheme of corpus callosum (CC) in follow-up data for acquiring follow-up whole CC (red) and its subsections (blue, just listed callosal prefrontal subsection as an example).

Before the connectivity-based parcellation, we first obtained the 3D CC in native space by using a semiautomatic procedure accomplished with Advanced Normalization Tools (ANTs) (Tustison et al., 2014) (Figure 1B): (1) the callosal mask in standard space was extracted from International Consortium for Brain Mapping (ICBM) atlas, (2) the FA template (FMRIB58_FA 1mm) in standard space was registered to native FA map using SyN registration algorithm (which combines affine and non-linear transformation) and the transform was saved, and (3) the callosal mask in native space was obtained by warping the callosal mask in standard space with the transform. Afterward, each native callosal mask was visually examined by an experienced radiologist who was blinded to the patient disease status and was manually adjusted and then used to locate the tracking seed.

### T1-Weigthed Imaging Preprocessing and Target Definition

T1-weighted images were preprocessed by ANTs, including intensity inhomogeneity correction (N4 bias correction), brain tissue extraction, SyN diffeomorphic image coregistration, and tissue segmentation, which has been proven to have superior performance over the FreeSurfer pipeline (Tustison et al., 2014). Afterward, a total of 62 cortical segmentations were obtained by employing the Desikan–Killiany–Tourville (DKT) cortical labeling protocol (Klein and Tourville, 2012) for each subject (Figure 1A). These segmentations were thresholded with 0.5 to rid white matter tissue and then binarized as binary mask. Of note, the premotor gyrus and prefrontal gyrus were separated from the superior frontal gyrus defined by the DKT atlas with the use of standard premotor gyrus in the Anatomical Automatic Labeling (AAL) atlas [Figure 1A,(b)]. As such, 64 binary cortical segmentations were acquired and transformed to diffusion space. Afterward, these segmentations were merged into five distinct segments (prefrontal, premotor, motor, somatosensory, and temporal–parietal–occipital segment) (Zhang et al., 2017) [Figure 1A,(d) and Table 1] to serve as the tracking targets.

**TABLE 1:** The callosal–cortical correspondence.

Callosal subsections	Cortical segmentations
Prefrontal	Bilateral caudal middle frontal, bilateral lateral orbitofrontal, bilateral medial orbitofrontal, bilateral pars opercularis, bilateral pars orbitalis, bilateral pars triangularis, bilateral rostral middle frontal, and bilateral prefrontal*
Premotor	Bilateral premotor*
Motor	Bilateral paracentral and bilateral precentral
Somatosensory	Bilateral postcentral
Temporal–parietal–occipital	Bilateral cuneus, bilateral entorhinal, bilateral fusiform, bilateral inferior parietal, bilateral inferior temporal, bilateral lateral occipital, bilateral lingual, bilateral middle temporal, bilateral pericalcarine, bilateral precuneus, bilateral superior parietal, bilateral superior temporal, bilateral supramarginal, and bilateral transverse temporal

Note. *These cortical segmentations were generated from the superior frontal gyrus defined by the Desikan–Killiany–Tourville (DKT) atlas.

### Connectivity-Based Parcellation

**FIGURE 2:**
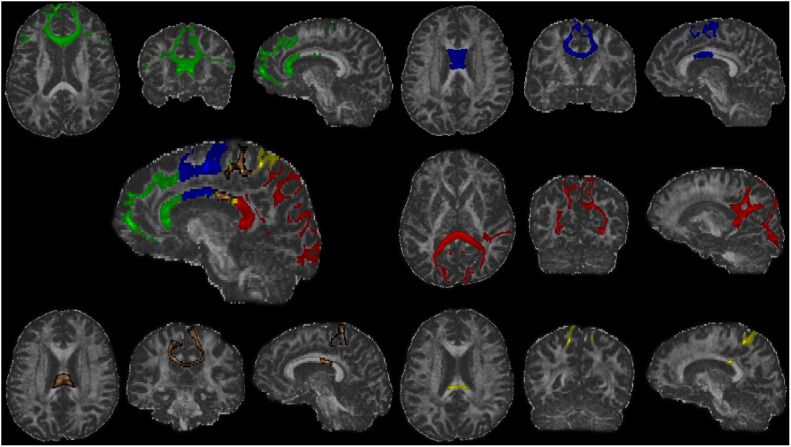
The fiber tracts of each callosal subsections (one representative subject). Green, the prefrontal subsection tract; blue, the premotor subsection tract; orange, the motor subsection tract; yellow, the somatosensory subsection tract; red, the temporal–parietal–occipital subsection tract.

After the definition of seed and targets, connectivity-based parcellation was performed in FSL. First, a probable fiber orientation estimation was executed in BedpostX, which stands for Bayesian Estimation of Diffusion Parameters Obtained using Sampling Techniques, and Markov Chain Monte Carlo sampling was ran to build up distributions on diffusion parameters at each voxel, thus modeling crossing fibers (Behrens et al., 2007). Then, based on the estimated fiber orientations, connectivity distributions between seed (3D callosal region) and target regions (five distinct cortical segments) were generated using FSL’s “Probtrackx” tool as displayed in Figure 2 with 5,000 samples per voxel, a step length of 0.5 mm, a curvature threshold of 0.2, and max number of steps per sample of 2,000. After tractography, each voxel in CC was classified into five classes according to the cortical region they mostly connected to (winner take all) (Behrens et al., 2003).

To make the data obtained from baseline and follow-up comparable, we segmented the follow-up CC as follows (executed in ANTs) (Figure 1C): (1) the baseline individual FA map was registered to the follow-up FA map and the transform was saved and (2) the follow-up CC and its subsections were acquired separately by warping baseline CC and its subsections with the transform.

### Imaging Metrics Calculation

The mean FA and MD values for CC and its subsections were extracted. The mean volumes of CC and its parcellated subsections were calculated as follows:

$$\text{Volume}\left({\text{cm}}^{3}\right)=\mathrm{Voxel}\mathrm{number}\times \mathrm{Imaging}\text{resolution}\left({\text{mm}}^{3}\right)\times 10^{-3}$$

Specifically, the total intracranial volume (TIV) of each subject was acquired as a covariate in the following volumetric regression model for eliminating individual morphological heterogeneity, and the cortical volumes of each person’s five cortical targets were calculated for illustrating the cortical structural alterations. First, the voxel number of binarized whole brain cortical tissue, and each cortical target was extracted, and then, the volume was calculated as stated above.

### Statistical Analysis

Demographic and clinical variables between groups were analyzed in Statistical Product and Service Solutions (SPSS version 23.0). The normal distribution of data was tested by Kolmogorov–Smirnov test. Differences between groups were analyzed with two-sample *t*-test, paired *t*-test, Pearson chi-square, or non-parametric tests appropriately. *P* < 0.05 was regarded as statistically significant.

The ROI-based analyses for FA, MD, and volume between PD at baseline (PD-BL) and NC were conducted by general linear model (GLM) with age, gender, and education as covariates. TIV was additionally regressed out in the volumetric group comparisons, and the paired *t*-test was employed for the comparisons between PD-BL and PD follow-up (PD-F). *P* < 0.05 was regarded as statistically significant for whole CC, while *p* < 0.01 (corrected by Bonferroni correction for multisubsection comparisons, *p* < 0.05/5) was regarded as statistically significant for each callosal subsection.

The relationships between specific metrics of different callosal subsections and clinical domains were evaluated by stepwise linear regression model at baseline and follow-up, respectively, and the relationships between baseline structural metrics and clinical annualized change were analyzed. Specifically, each clinical domain *z*-score was used as dependent variable; age, gender, and education as force-entered covariates in the first block; and imaging metrics (FA, MD, and volume, each run independently) of the five callosal subsections as stepwise-entered independent variables in the second block with enter *p*-value = 0.05 and remove *p*-value = 0.1 (Bledsoe et al., 2018). When the originally introduced variable becomes statistically insignificant due to the introduction of a later variable, it will be removed; thus, only the significant variables would remain in the last calculated model, and *p* < 0.05 was used to evaluate the significance of each constructed model.

## Results

### Demographic and Clinical Variables

Demographic and clinical variables are shown in Table 2. No significant difference was observed in basic demographic information (including age, *p* = 0.366; gender, *p* = 0.094; and education, *p* = 0.080, respectively) between PD-BL and NC. For clinical assessments, PD patients at baseline showed no difference in MMSE scores (*p* = 0.325) and ESS scores (*p* = 0.926), while HAMD scores (*p* < 0.001) and HAMA scores (*p* = 0.003) were increased compared with NC.

**TABLE 2:** Demographic and clinical variables.

Clinical variables	NC	PD-BL	PD-F	*P*-value		
				PD-BL/NC	PD-F/NC	PD-BL/PD-F
Num	82	39	39	–	–	–
Age (years)	59.19 ± 6.60	60.45 ± 8.09	62.10 ± 8.25	0.366^a^	**0.038**^a^*	–
Age range (years)	46–77	44–82	45–84	–	–	–
Gender (male/female)	47/35	16/23	16/23	0.094^b^	0.094^b^	–
Education (years)	9.57 ± 4.37	8.05 ± 4.53	8.05 ± 4.53	0.080^a^	0.080^a^	–
Disease duration (years)	–	3.40 ± 2.65	4.97 ± 2.71	–	–	–
LED	–	269.23 ± 232.58	460.18 ± 267.34	–	–	**<0.001**^c^*
UPDRS III	–	23.33 ± 13.39	19.69 ± 13.43	–	–	**0.011**^d^*
H-Y	–	2.5 (1.5–3)	2.5 (1–4)	–	–	**0.018**^d^*
HAMD	2.08 ± 2.47	5.13 ± 3.71	5.97 ± 4.65	**<0.001**^a^*	**<0.001**^a^*	0.283^c^
HAMA	3.01 ± 3.50	4.59 ± 3.39	7.13 ± 5.04	**0.003**^a^*	**<0.001**^a^*	**0.002**^c^*
ESS	4.98 ± 3.99	5.13 ± 4.63	5.61 ± 21.17	0.926^a^	0.665^a^	0.577^c^
PDSS	–	131.64 ± 20.91	122.77 ± 3.39	–	–	**0.038**^c^*
MMSE	28.13 ± 2.03	27.38 ± 3.17	27.15 ± 2.82	0.325^a^	**0.028**^a^*	0.394^d^

The continuous variables were represented as mean ± SD, while the categorical variable (H-Y) was presented as median (range). NC, normal control; PD-BL, PD at baseline; PD-F, PD follow-up. ^*a*^Mann–Whitney *U*-test. ^*b*^Pearson chi-square. ^*c*^paired *t*-test. ^*d*^Wilcoxon test. *Significant result with p < 0.05.

After the mean time interval of 21 months, PD-F patients showed higher HAMD scores (*p* < 0.001) and HAMA scores (*p* < 0.001) and lower MMSE scores (*p* = 0.028) than baseline NC. Direct comparisons of PD-BL and PD-F revealed that PD-F patients exhibited increased LED (*p* < 0.001), H-Y (*p* = 0.018), and HAMA scores (*p* = 0.002) and decreased UPDRS III (*p* = 0.011) and PDSS (*p* = 0.038) than PD-BL.

### Microstructural Changes of CC and Its Subsections in PD Patients

**FIGURE 3:**
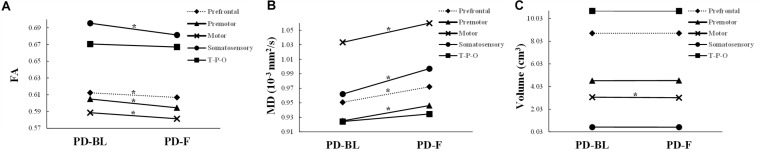
The structural alterations of FA **(A)**, MD **(B)**, and volume **(C)** in PD patients with disease evolution. PD-BL, PD at baseline; PD-F, PD follow-up. T-P-O, temporal-parietal-occipital subsection. *Significant result.

No significant microstructural alterations in the whole CC or its subsections were observed between PD-BL and NC (Table 3). Within the paired *t*-test for longitudinal data, significant reductions of FA and increments of MD in the whole CC (both *p* < 0.001) and its anterior part, including prefrontal (both *p* < 0.001), premotor (*p* < 0.001 and *p* = 0.001, respectively), motor (*p* = 0.003 and *p* < 0.001, respectively), and somatosensory (*p* = 0.001 and *p* = 0.002, respectively) subsections, were observed in PD-F when comparing with PD-BL (Figure 3 and Table 3).

**TABLE 3:** Group comparisons of callosal structural metrics.

Imaging metrics	Subsections	NC	PD-BL	PD-F	*p*-Values	
					PD-BL/NC	PD-BL/PD-F
***Part 1 Group comparisons of callosal subsections***						
FA	Prefrontal	0.62 ± 0.02	0.61 ± 0.03	0.61 ± 0.03	0.158	<0.001^a^*
	Premotor	0.61 ± 0.04	0.60 ± 0.04	0.59 ± 0.04	0.361	<0.001^b^*
	Motor	0.59 ± 0.03	0.59 ± 0.04	0.58 ± 0.04	0.782	0.003^a^*
	Somatosensory	0.67 ± 0.08	0.70 ± 0.08	0.68 ± 0.08	0.079	0.001^a^*
	T-P-O	0.68 ± 0.02	0.67 ± 0.03	0.67 ± 0.03	0.471	0.016^a^
MD(10^–3^ mm^2^/s)	Prefrontal	0.93 ± 0.05	0.95 ± 0.07	0.97 ± 0.08	0.057	<0.001^a^*
	Premotor	0.89 ± 0.06	0.93 ± 0.07	0.95 ± 0.08	0.034	0.001^a^*
	Motor	1.01 ± 0.06	1.03 ± 0.08	1.06 ± 0.08	0.375	<0.001^a^*
	Somatosensory	1.00 ± 0.26	0.96 ± 0.15	1.00 ± 0.15	0.254	0.002^b^*
	T-P-O	0.91 ± 0.05	0.92 ± 0.06	0.93 ± 0.07	0.747	0.026^a^
Volume (cm^3^)	Prefrontal	8.62 ± 1.10	8.74 ± 1.06	8.74 ± 1.12	0.585	0.941^a^
	Premotor	4.37 ± 0.90	4.55 ± 0.94	4.56 ± 0.96	0.614	0.795^a^
	Motor	3.10 ± 0.50	3.09 ± 0.54	3.05 ± 0.55	0.900	0.004^a^*
	Somatosensory	0.52 ± 0.34	0.45 ± 0.32	0.45 ± 0.32	0.293	0.518^a^
	T-P-O	10.63 ± 1.58	10.71 ± 1.89	10.71 ± 1.99	0.696	0.908^a^
***Part 2 Group comparisons of whole CC***						
FA	Whole CC	0.64 ± 0.02	0.63 ± 0.03	0.63 ± 0.03	0.222	<0.001^a^*
MD(10^–3^ mm^2^/s)	Whole CC	0.93 ± 0.05	0.95 ± 0.06	0.96 ± 0.07	0.150	<0.001^a^*
Volume (cm^3^)	Whole CC	27.24 ± 2.93	27.55 ± 3.18	27.50 ± 3.43	0.968	0.677^a^

NC, normal controls; PD-BL, PD at baseline; PD-F, PD follow-up; T-P-O, temporal–parietal–occipital subsection; CC, the whole 3D corpus callosum. The comparisons of PD-BL/NC were conducted by general linear model. FA and MD comparisons were regressed age, gender, and education out, while in volumetric comparisons, the total intracranial volume was additionally regressed out. The comparisons of PD-BL/PD-F were conducted by paired ^a^*t*-test or ^b^Wilcoxon test. Bonferroni correction (*p* < 0.05/5 = 0.01) was performed for Part 1, while *p* < 0.05 was regarded as statistically significant for Part 2. All variables were represented as mean ± SD; *Significant result with p < 0.01 for Part 1, and p < 0.05 for Part 2.

To alleviate the effect of partial volume contamination on FA and MD, we did an eroding procedure on each CC subsection as well as the whole CC mask. The details were as follows: the whole CC mask and its each subsection mask were eroded by one edge voxel; the mean imaging metrics of each eroded callosal mask were calculated and analyzed by the previous statistical approaches. Of note, as the volume of somatosensory subsection was very small (NC, 0.52 ± 0.34 cm^3^; PD-BL, 0.45 ± 0.32 cm^3^; PD-F, 0.45 ± 0.32 cm^3^) and all somatosensory subsection masks were located at relatively the central part of CC, we did not perform the eroding procedure on this subsection. The results are shown in Supplementary Table S1, in which we could find that the changing trend was similar with our former results.

### Macrostructural Changes of CC and Its Subsections in PD Patients

No volumetric alterations of whole CC or its subsections were detected in PD-BL compared with NC (Table 3). However, during disease course, the volume of motor subsection (*p* = 0.004) was decreased in PD-F patients when comparing with PD-BL (Figure 3 and Table 3).

### Cortical Macrostructure Alterations in PD Patients

There was no difference in cortical volume either between PD-BL and NC or between PD-BL and PD-F at whole cortex level or segment level.

### Relationships Between Callosal Imaging Metrics and Clinical Domains in PD

The FA of temporal–parietal–occipital subsection (*p* = 0.046, *R*^2^ = 0.307) and the volume of motor subsection (*p* = 0.016, *R*^2^ = 0.344) were correlated with mood domain at baseline, and the MD of somatosensory subsection (*p* = 0.041, *R*^2^ = 0.272) was associated with motor domain at follow-up (Table 4). However, the baseline structural metrics were found having no significant relationship with the annualized clinical change. These showed that the callosal subsections were supposed to possess specific associations with corresponding clinical performance in PD.

**TABLE 4:** The significant results of regression models.

Clinical domains	Callosal subsection with significant association	Adjusted *R*^2^	*F*	*p*-Values
**Baseline**				
Mood	FA (T-P-O subsection)	0.307	5.206	**0.002^∗^**
	Volume (motor subsection)	0.344	5.977	**0.001^∗^**
**Follow-up**				
Motor	MD (somatosensory subsection)	0.272	4.552	**0.005^∗^**

Regression models were constructed between structural metrics and clinical domains at baseline and follow-up, respectively, and the relationships between baseline structural metrics and clinical annualized change were analyzed. Regression models were controlled for age, gender, and education. T-P-O, temporal−parietal−occipital subsection. *Significant result with p < 0.05.

## Discussion

With longitudinal design, our study demonstrated the structural characteristics of callosal subsections in PD over time. The main findings included the following: (1) no significant macro/microstructural alterations were found in PD-BL when compared to NC; (2) after a mean time interval of 21 months, PD-F showed significantly decreased volume in motor subsection, decreased FA, and increased MD in the whole CC and its subsections (except the temporal–parietal–occipital subsection) compared with PD-BL; (3) the cortical volume did not show any significant differences between groups or during disease course; and (4) the baseline FA of temporal–parietal–occipital subsection, as well as the volume of motor subsection, was correlated with the mood domain, and the follow-up MD of somatosensory subsection was associated with motor domain.

### The Early Preserved Callosal Macro/Microstructure Was Progressively Disrupted in PD

At baseline, no macro/microstructural alterations in PD-BL subjects were detected. Interestingly, during disease course, decreased FA and increased MD of whole CC and its subsections (except temporal–parietal–occipital subsection) and decreased volume of motor subsection were observed. The micro- and macrostructures of CC and its subsections in PD-BL, which was at a relatively early stage, may be fairly preserved and, along the disease progression, became disrupted. Consistent to our results, Taylor et al. (2018) revealed that over 1-year follow-up, PD patients had significantly stronger decline in FA and stronger increase in MD in the callosal region compared with NC. Racheal et al. (Guimaraes et al., 2018) suggested that PD patients showed microstructural alterations of CC only in late stage. However, Gattellaro et al. (2009) conducted a cross-sectional study and reported that the microstructure of the callosal genu was disrupted in PD at an early stage, which may be due to the sample heterogeneity of PD patients or the relatively small sample size of their study. In general, these findings suggested that the early preserved callosal macro-/microstructure was progressively disrupted in PD.

Parkinson’s disease is a progressive neurodegenerative disorder, the disease severity and the brain pathological alterations of which are progressively aggravated with disease evolution (Taylor et al., 2018). As Braak et al. (2003) suggested, the pathological process underlying PD was an ascending course, from the brain stem ascending to the neocortex. At a relatively early stage, CC was fairly preserved and, with disease progression, gradually became disrupted, which was consistent with the undamaged cortical macrostructure found in our subjects. The symptoms of our subjects were relatively mild, which might stay at a low pathological stage. Consistent with our findings, the former studies reported no cortical change in early PD when directly compared with NC (Ibarretxe-Bilbao et al., 2012; Tessitore et al., 2012). Although Ibarretxe-Bilbao et al. (2012) found that the total cortical volume in PD was decreased during the disease course, their following mean time interval (35 months) was longer than ours. Therefore, the disease duration may be an indicator reflecting the underlying pathological change in a degree; thus, longer-term follow-up was still needed to validate these findings.

It was worth mentioning that though the motor subsection was atrophied, the motor symptoms were alleviated during disease course. The beginning of drug management or the increment of dosage in PD-F may account for it, which further indicated that the patients who followed with a medical disease duration (mean time interval of 21 months) might benefit from anti-parkinsonian therapy with brain reorganization, though the patients were required to withdraw all anti-parkinsonian medicine overnight. Thus, on the lasting influence of anti-parkinsonian therapy, the clinical evaluation cannot truly reflect the disease progression. Instead, the disrupted structure of the CC and its subsections during the longitudinal observation, detected by this study, provided such objective evaluation.

### The Topographic Organization Feature of CC in PD

In the specific observation of the structural characteristics of CC and its subsections, different callosal subsections showed the distinct appearance during disease evolution. It has been well established that the anterior CC (mostly similar to the prefrontal, premotor, motor, and somatosensory subsections in the present study) has high concentration of small and unmyelinated axons in the structure (Aboitiz et al., 1992), which appears to be selectively vulnerable to develop abnormal proteinaceous aggregations in PD (Braak et al., 2006). In agreement with our findings, structural disruptions in these callosal subsections were widely reported (Fling et al., 2016; Bledsoe et al., 2018). Specifically, one study exhibited disrupted white matter in callosal regions connecting bilateral premotor, motor, and somatosensory cortices (Fling et al., 2016), while another investigation revealed that the volume in the mid-anterior and central callosal regions was reduced (Goldman et al., 2017). Therefore, this longitudinal observation, by employing connectivity-based parcellation, further demonstrated that the topographic organization is an important feature of CC (Caille et al., 2005; Fabri et al., 2014), which was widely and progressively disrupted under PD pathology, e.g., direct abnormal protein aggregation or indirect neurodegeneration.

### The Specific Relationship Between Callosal Subsections and Clinical Performance in PD

It was noticeable that regional callosal characteristics showed significant associations with clinical performance such that the FA of temporal–parietal–occipital subsection as well as the volume of motor subsection was found to be correlated with the mood domain at baseline, while the MD of somatosensory subsection was associated with motor domain at follow-up. The bodily experience would affect previous emotional processing as well as ongoing and subsequent emotional processing (Sasaki et al., 2015). Transcranial magnetic stimulation analysis showed that the negative emotion could enhance long-term potentiation like plasticity in the human primary motor cortex over time and space (Koganemaru et al., 2012). Given the connection of the callosal region to the corresponding motor cortex, the shrunken motor subsection would contribute to the emotional disturbances in PD as well as the temporal–parietal–occipital callosal subsection, which would participate in the emotional regulation through influencing information transfer to the temporal, parietal, and occipital cortices, which were reported to have interactions with emotional processing (Feldmann et al., 2008; Liang et al., 2016). The findings of significant relationships between mood domain and FA value in the temporal–parietal–occipital subsection in which structural differences were not detected highlight the multifaceted and complex nature of these relationships.

Besides, a mass of evidence indicates that the perceptional function in PD patients interacts with the motor output (Conte et al., 2013). Also, the somatosensory area, which is the main sensory administrative point, has been found to be underactive in PD (Foki et al., 2015), and most of which are involved in movement-related symptoms (Conte et al., 2013). Incorrect sensory signals would influence the preparation and execution of voluntary movement. The integrity of somatosensory callosal subsection was disrupted, which would affect the information transfer to somatosensory regions and thereby play a role in the maintenance of normal motor function. The different relationships found at baseline and follow-up may be because non-motor symptoms played an important role at a relatively early stage (Bassetti, 2011), like mood showed the significant between-group differences at baseline, which accounted for the early detection of relationship between structural metrics and mood domain. In combination with these findings, we thereby concluded that the callosal regions would be specifically linking to the clinical performance in PD.

Technologically, this study differs in several methodologic aspects from former callosal studies (Witelson, 1989; Lebel et al., 2010; Zhang et al., 2017). The most important strength of our study was the usage of connectivity-based parcellation that separated CC into functionally distinct subsections for better defining boundaries between each two subsections. Besides, the evaluation of CC was 3D, which decreased the artificial bias and improved the signal-to-noise ratio (Lebel et al., 2010).

We acknowledged potential study limitations such that the longitudinal sample size of our study was relatively small; thus, it should be cautious to extend our findings to some specific PD populations. Another critical evaluation of future work is the lack of longitudinal observation in NC, which could take the advantages of further elucidating relationships between longitudinal callosal changes and disease status. The following time interval of our subjects was relatively short, which would restrain our findings; studies with longer-term follow-up are still needed to clarify such complex nature of disease. Future histopathologic studies may help verify the underlying structural nature of the imaging metrics and broaden its use.

## Conclusion

The longitudinal findings, in the present study, give a comprehensive evaluation of the dynamic structural alterations in CC, and its connectivity-specific subsections with their macro-/microstructure remain fairly preserved in PD at a relatively early stage and progressively disrupted during the disease course. Besides, the different callosal subsections possess specific associations with clinical performance and are closely associated with non-motor dysfunctions at a relatively early stage in PD.

## Data Availability Statement

The datasets presented in this article are not readily available because the data are not publicly available due to their containing information that could compromise the privacy of research participants. Requests to access the datasets should be directed to zhangminming@zju.edu.cn.

## Ethics Statement

The studies involving human participants were reviewed and approved by the Medical Ethic Committee of The Second Affiliated Hospital of Zhejiang University School of Medicine. The patients/participants provided their written informed consent to participate in this study.

## Author Contributions

JW: conceptualization and writing–original draft. TG: visualization, investigation, and methodology. CZ: visualization and investigation. XG: conceptualization and methodology. TG and BZ: resources and investigation. MX, QG, PH, ZS, JP, YY, and JT: resources. XX: writing–review and editing. MZ: writing–review and editing and supervision. All authors contributed to the article and approved the submitted version.

## Conflict of Interest

The authors declare that the research was conducted in the absence of any commercial or financial relationships that could be construed as a potential conflict of interest.

## Footnotes

1 http://www.nitrc.org/projects/panda/

2 http://www.fmrib.ox.ac.uk/fsl/

3 http://trackvis.org/dtk/
