# Supplementary material for: Longitudinal Macro/Microstructural Alterations of Different Callosal Subsections in Parkinson’s Disease Using Connectivity-Based Parcellation
Source: Front Aging Neurosci. 2020 Nov 4;12:572086. doi: 10.3389/fnagi.2020.572086 (PMC7672016; doi:10.3389/fnagi.2020.572086)
Supplement: Supplementary file 1 [file Table_1.docx]

**Table S1** Group comparisons of shrunken callosal structural metrics.

| Imaging metrics | Subsections | NC | PD-BL | PD-F | p values | |
| --- | --- | --- | --- | --- | --- | --- |
|  |  |  |  |  | PD-BL/NC | PD-BL/PD-F |
| ***Part1 Group comparisons of shrunken callosal subsections*** | | | | | | |
| FA | Prefrontal | 0.73±0.02 | 0.73±0.02 | 0.73±0.02 | 0.762 | 0.399^a^ |
|  | Premotor | 0.72±0.03 | 0.72±0.03 | 0.71±0.03 | 0.841 | **<0.001^a*^** |
|  | Motor | 0.73±0.03 | 0.73±0.04 | 0.71±0.04 | 0.341 | **<0.001^a*^** |
|  | T-P-O | 0.78±0.02 | 0.78±0.02 | 0.78±0.02 | 0.122 | 0.967^b^ |
| MD  (10^-3^ mm^2^/s) | Prefrontal | 0.84±0.03 | 0.84±0.03 | 0.84±0.04 | 0.946 | 0.163^b^ |
|  | Premotor | 0.82±0.04 | 0.84±0.03 | 0.84±0.03 | 0.054 | 0.627^a^ |
|  | Motor | 0.88±0.03 | 0.89±0.04 | 0.90±0.06 | 0.849 | 0.013^a^ |
|  | T-P-O | 0.79±0.03 | 0.80±0.04 | 0.80±0.04 | 0.659 | 0.558^b^ |
| ***Part2 Group comparisons of shrunken whole CC*** | | | | | | |
| FA | Whole CC | 0.76±0.02 | 0.75±0.02 | 0.75±0.02 | 0.474 | **0.019^a*^** |
| MD  (10^-3^ mm^2^/s) | Whole CC | 0.82±0.03 | 0.82±0.03 | 0.83±0.04 | 0.489 | 0.173^a^ |

NC, Normal controls; PD-BL, PD at baseline; PD-F, PD follow-up; T-P-O, temporal-parietal-occipital subsection; CC, the whole 3D corpus callosum. The comparisons of PD-BL/NC were conducted by General Linear Model. FA and MD comparisons were regressed age, gender and education out. And the comparisons of PD-BL/PD-F were conducted by paired t-test (a) or Wilcoxon test (b). Bonferroni correction (p<0.05/5=0.01) was performed for Part1, while p<0.05 was regarded as statistically significant for Part2. All variables were represented as mean±SD; * denotes the significant result.
